# Modeling the effect of climatic conditions and topography on malaria incidence using Poisson regression: a Retrospective study in Bannu, Khyber Pakhtunkhwa, Pakistan

**DOI:** 10.3389/fmicb.2023.1303087

**Published:** 2024-01-15

**Authors:** Ijaz Ul Haq, Zafar Mehmood, Gausal Azam Khan, Bushra Kainat, Bilal Ahmed, Jahan Shah, Amtul Sami, Muhammad Subhan Nazar, Jielian Xu, He Xiang

**Affiliations:** ^1^Department of Public Health & Nutrition, The University of Haripur, Haripur, Khyber Pakhtunkhwa, Pakistan; ^2^Department of Maths, Stats & Computer Science, The University of Agriculture Peshawar, Peshawar, Khyber Pakhtunkhwa, Pakistan; ^3^Department of Clinical Nutrition, College of Applied Medical Sciences, King Faisal University, Al Ahsa, Saudi Arabia; ^4^School of Pharmacy, Nanjing Medical University, Nanjing, Jiangsu, China; ^5^Department of Social Medicine and Health Education, School of Public Health, Nanjing Medical University, Nanjing, Jiangsu, China; ^6^Department of Health Biotechnology, Women University, Swabi, Khyber Pakhtunkhwa, Pakistan; ^7^Department of Clinical Nutrition, The Affiliated Jiangning Hospital of Nanjing Medical University, Nanjing, Jiangsu, China

**Keywords:** malaria, incidence, topography, rainfall, humidity, temperature

## Abstract

**Background:**

Malaria has been identified as a crucial vector-borne disease around the globe. The primary aim of this study was to investigate the incidence of malaria in the district of Bannu and its relationship with climatic conditions such as temperature, rainfall, relative humidity, and topography.

**Methods:**

Secondary data were obtained from the metrological office and government hospitals across the district for 5 years (2013–2017). A Poisson regression model was applied for the statistical analysis.

**Results and discussion:**

The number of reported cases of malaria was 175,198. The regression analysis showed that temperature, relative humidity, and rainfall had a significant association (*p* < 0.05) with malaria incidence. In addition, the topographic variables were significantly associated (*p* < 0.05) with malaria incidence in the region. The percent variation in the odds ratio of incidence was 4% for every unit increase in temperature and 2% in humidity. In conclusion, this study indicated that the temperature, humidity, rainfall, and topographic variables were significantly associated with the incidence of malaria. Effective malaria control and interventions integrated with climatic factors must be considered to overcome the disease burden.

## Introduction

In Pakistan, malaria is endemic, affecting millions of lives per year (Khan et al., 2023). According to the World Health Organization (WHO), 3.4 million suspected cases were reported between January and August 2022 in Pakistan (WHO, 2022). The Directorate of Malaria Control reported 1.7 million confirmed malaria cases, comprising 32% *Plasmodium falciparum (P. falciparum)* cases, 67% *Plasmodium vivax (P. vivax)* cases, and 1% mixed infections during 2022 (DMC, 2022). Pakistan’s National Malaria Control Program reported that a six-fold increase in *P. falciparum* Malaria has been observed in the last decade, comprising 42% of all malaria cases reported in the country (Yasinzai and Kakarsulemankhel, 2008). Moreover, malaria is the fourth-largest cause of death among infectious diseases in Pakistan. It accounts for more than 95% of the total regional malaria burden in Afghanistan, Somalia, Sudan, and Yemen (Jahan et al., 2019). The National Annual Parasite Incidence (API), averaging 1.66 per 1,000 populations suffering from malaria, has been reported in Pakistan (Jahan et al., 2019).

An increase in malaria incidence has been observed since 2004 in various Southeast Asian countries (McCutchan et al., 2008). According to the WHO, 40% of the world’s population is at risk of developing malaria (Patel et al., 2004). Other studies have shown that parasitic malaria infection has increased since 2015 (Dhiman, 2019). An estimated 219 million people were infected with malaria, which caused 435,000 deaths in 2017 worldwide. The morbidity and mortality burdens resulted from global effort and research to improve malaria prevention, diagnosis, and treatment for more than a century. The global mortality rate of malaria ranges from 0.3 to 2.2%, and in regions with tropical climates, the mortality rate ranges from 11 to 30% with severe forms of malaria (Talapko et al., 2019). According to the WHO, children under 5 years of age account for nearly 80% of all malaria fatalities.

Changes in the temporal and spatial temperature, precipitation, and humidity under different climate change scenarios affect the biology and ecology of vectors and intermediate hosts, thus creating the risk of disease transmission (Lindsay and Birley, 1996). The higher latitude results in temperature changes, leading to early signs of changing distribution of vector-borne diseases (Bergquist et al., 2021). The cause of vector-borne diseases (V.B.D.s) are arthropods that transmit viruses among humans. Examples of V.B.D.s are dengue and malaria (mosquito-borne), Lyme disease (tick-borne), and leishmaniosis (transmitted by sandflies) (Ogden et al., 2013). Human travel and transport, animal migration, environmental changes to habitats, and vector range expansion caused by global trade and climate change are all forms of animal-borne diseases within a population (Nah et al., 2016). Climatic factors such as precipitation, wetness, and hotness substantially affect the occurrence of malaria caused by parasites. Higher temperatures can reduce the extrinsic incubation period, potentially increasing the likelihood of malaria transmission (Jackson et al., 2010).

Topography also has a high role in spreading vector-borne diseases. Incidence of malaria and topography have a positive relationship; i.e., the risk of malaria increases in highlands compared to lowlands (Atieli et al., 2011). The complexity of the terrain and the geography of the highlands contribute to the diversity of vector abundance and the potential for malaria transmission, resulting in vector survival at different heights (Minakawa et al., 2002).

Malaria is a vector-borne disease caused by the bite of a female Anopheles mosquito that causes life-threatening infection in the human bloodstream (Patel et al., 2004). In severe cases, malaria leads to calamitous complications, which have significant and widespread consequences in a community (Patel et al., 2004). The disease is caused by infection with a parasitic unicellular organism of the genus Plasmodium, injected through the bite (Snyder and History, 1993). Traditionally, four types of Plasmodium, including *P. falciparum, P. vivax, Plasmodium ovale*, and *Plasmodium malariae*, are known to cause disease in humans (McCutchan et al., 2008). The most common symptoms include high fever, chills, vomiting, and jaundice, especially associated with hemolytic anemia, hemoglobinuria, and various stages of thrombocytopenia (Coelho et al., 2013). Cerebral malaria and kidney failure are the most severe complications (Sajjanar et al., 2013).

Pakistan’s highest APIs are reported most in Federally Administered Tribal Areas (FATA), Baluchistan, and Khyber Pakhtunkhwa (K.P.), where 66 districts and agencies are divided into high endemicity stratum (API) > 5 per 1,000). In K.P., malaria incidence is an alarming situation (Ullah Khan, 2018). There is a connection between malaria and climate change as the outbreak of malaria has been observed in the aftermath of heatwaves in the past (Noureen et al., 2022). Several factors have contributed to the increase in malaria rates over the years, including warm autumn (which also increases the duration of infection), the emergence of chloroquine resistance worldwide (Shah et al., 1997), and a steady decline in vector control activities (Jahan et al., 2019).

Designing effective control and prevention strategies requires considering human and climate factors, such as rainfall, age, gender, migration, type, and housing location, that may contribute to local malaria transmission (Snow et al., 1997; Thompson et al., 1997). Spatial variability and chronology in malaria transmission require timely identification of significant risk factors to implement targeted control measures (Klinkenberg et al., 2004). In Pakistan, malaria exhibits an unusual distribution. The spread of malaria follows a seasonal pattern in Pakistan, with high rates occurring in September and October. Another peak is observed at the beginning of summer. Currently, there are no available data that analyze the modeling impact of climate and topography on malaria in Pakistan. Therefore, we formulate the research questions “How do climatic conditions and topography contribute to the incidence of malaria in the Bannu district of Khyber Pakhtunkhwa, Pakistan, and how can this relationship be quantified and modeled using Poisson regression?” Therefore, this study aimed to find the modeling effect of climatic conditions and topography on malaria incidence in Bannu, Pakistan. Creating accurate models to predict malaria and the impact of climate and topography is crucial for the identification of high-risk areas for timely intervention.

## Methods

### Study area and participants

**Figure 1:**
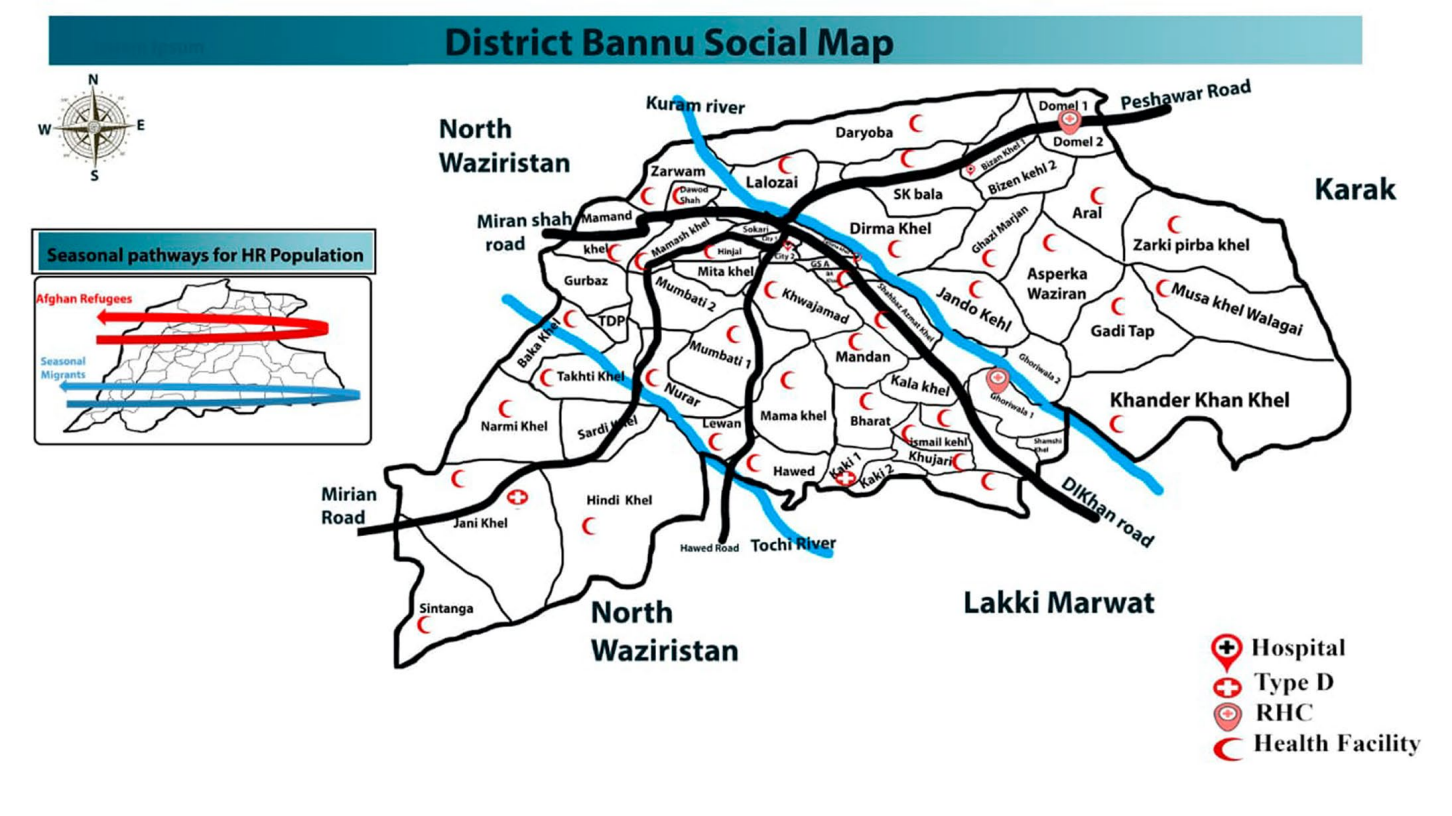
District Bannu Map according to health facilities. Source: District Health Office Bannu.

The current study was carried out in various parts of District Bannu, Khyber Pakhtunkhwa, Pakistan, which is surrounded by the rugged and arid mountain ranges of Koh-e-Hindukush and Koh-e-Sufaid. Bannu is located approximately 120 km southwest of Kohat, 140 km north of D. I. Khan, and approximately 190 km south of Peshawar. The entire area of this city covers 1,227 sq km. Bannu is situated between 32° 59′ and 33° 22′ north latitude and 70° 36′ and 71° 21′ east longitude. The region between the Kurram River and the Gambila River is known as the floodplain. In District Bannu, winter typically begins in the third week of November and lasts until the end of March, while the summer season spans from May to September. The estimated population of District Bannu is approximately 1,357,890. Health facility wise map has been shown (Figure 1).

Regarding the participants, we collected secondary data from health facilities and other offices. Our data included the incidence of malaria cases, topography, and climatic factors in accordance with the objectives of the study. This study adhered to the guidelines set forth in the Declaration of Helsinki. The ethical committee of the University of Agriculture Peshawar, Pakistan approved this study under IRB#05.

### Data collection

The area-wise data of the various zones of Bannu district included in the study were obtained from the Population Census of Pakistan (Pakistan Bureau of Statistics, 2023). Climatological data, including information on rainfall, temperature, and humidity, were obtained from the Regional Meteorological Office in Peshawar, Pakistan (Pakistan Meteorological Department, 2023). The secondary data included monthly rainfall measurements in millimeters, humidity levels in percentage, and temperature data in degree Celsius. The data on the number of individuals with malarial fever were collected from Khalifa Gulnawaz Hospital, Women and Children Hospital, the District Headquarters Hospital, basic health units, and dispensaries in Bannu district, Khyber Pakhtunkhwa, Pakistan. We included all malaria incident cases in the health facilities of Bannu district during the 5-year period (60 months), i.e., January 2013 to December 2017, for the study. Experienced statisticians and researchers checked the data for duplication, data entry errors, and other data quality issues. For this study, the case definition of malaria included individuals with a confirmed record of malaria who were not hospitalized, as well as individuals with hospitalization records indicating a diagnosis of malaria. In the health facilities, malarial parasite blood smear tests were conducted to diagnose malaria in patients.

### Statistical analysis

SPSS 22.0 (SPSS, Chicago, IL, United States) was used for the analysis. Time series analysis and Poisson regression analysis were applied for the malaria trend and association with other factors, respectively. The autoregressive distributed lag model (ADLM) was utilized to assess lag effects and to determine the R-squared and adjusted R-squared values for model fitting. Details of statistical models have been shown in Supplementary File 1. A value of *p* of <0.05 were considered significant.

## Results

The data on malaria were obtained from government hospitals and dispensaries in Bannu district from 2013 to 2017. The confirmed incidence of malaria at the outpatient department (O.P.D) section of the facilities and hospitals was the response (dependent) variable, and temperature, rainfall, humidity, and topography were predictors (independent) variables.

According to the sources of government hospitals in Bannu district, total cases of malaria occurrence were 175,198. The maximum temperature in the study was 42°C. Furthermore, maximum humidity and rainfall were 83% and 149 mm, respectively (Table 1).

**Table 1:** Mean (SD)/median(IQR) of climatic factors.

	Minimum	Maximum	Mean (SD)^a^/Median (IQR)^b^
Temperature (°C)	19	42	29.5 (7.4)^a^
Rainfall (mm)	0	149	30.5 (15.0)^b^
Humidity (%)	40	83	66.4 (10.9)^a^

### Malaria and rainfall

**Figure 2:**
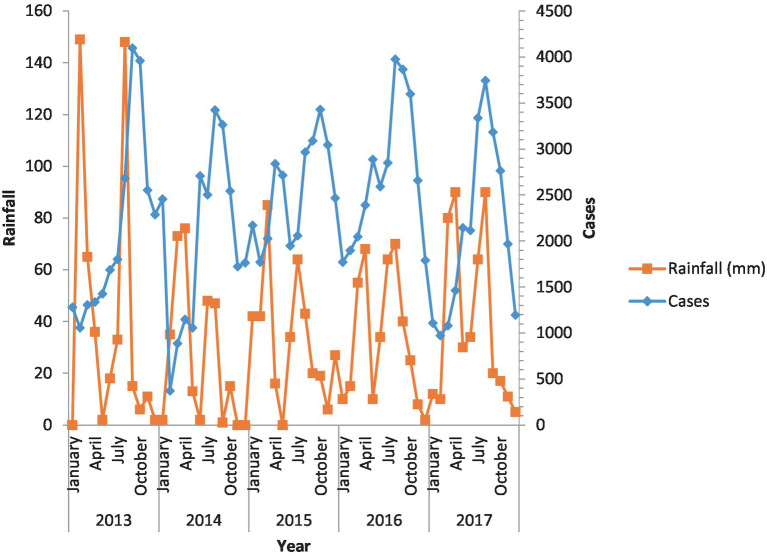
Incidence of malaria against rainfall. Malaria cases and rainfall over the 5 years have been shown.

Figure 2 depicts the incidence of malaria in relation to rainfall. Malaria incidence shows an increase with rising levels of rainfall. According to the regional weather forecasting department, the area experiences the highest recorded rainfall. This increased rainfall subsequently leads to higher parasitic density in the region, creating a favorable environment for the parasites during the onset of the rainy period.

### Malaria and temperature

**Figure 3:**
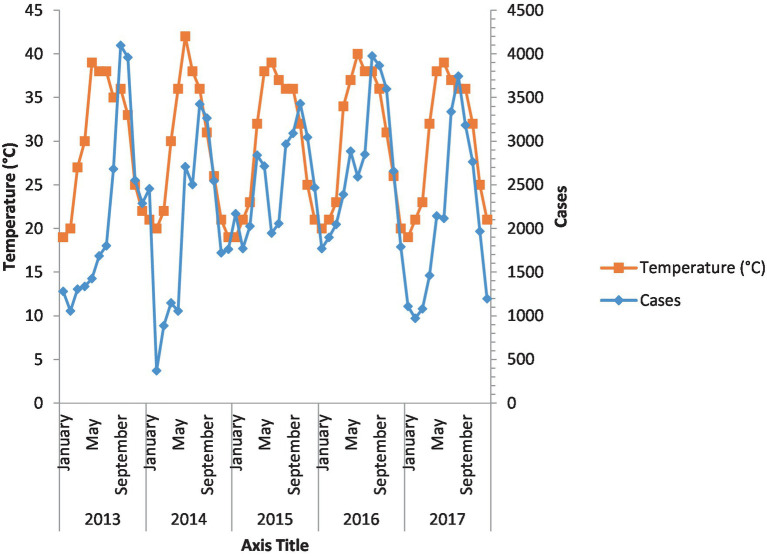
Incidence of malaria against temperature. Malaria cases and temperature over the 5 years have been shown.

Figure 3 shows the incidence of malaria against temperature. The cases are consistent over the 5 years. Cases of malaria increased with an increase in temperature over the years.

### Malaria and humidity

**Figure 4:**
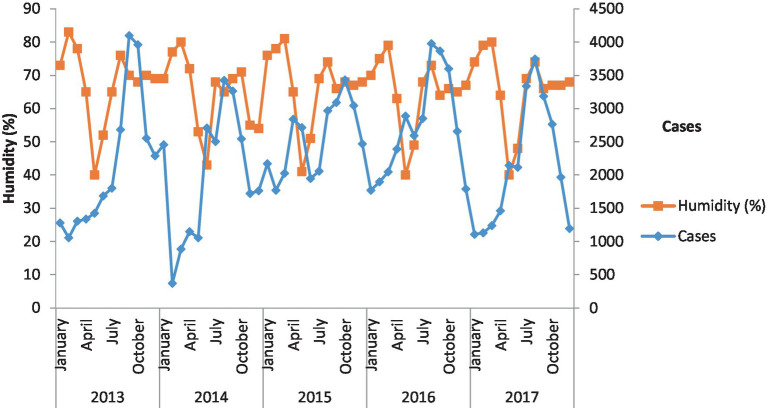
Incidence of malaria against humidity. Malaria cases and humidity over the 5 years have been shown.

Figure **4** shows the incidence of malaria against humidity. In the context of humidity, the cases of malaria are consistent. In 2013, 2014, and 2015, humidity increased malaria cases in the study area.

*Results of Poisson regression model*

**Log (prev) = Intercept + b**_**1**_**(Topo = 1) + b**_**2**_**(Topo = 2) + b**_**3**_**tem + b**_**4**_**rain + b**_**5**_**hum.**

**Prev** = exp(Intercept) * exp(b_1_(Topo =1)) * exp(b_2_(Topo = 2)) * exp(b_3_ tem) * exp(b_4_ rain) *exp(b_5_ hum).

**Prev** = exp(4.001) * exp(− 0.408 (Topo =1) * exp(0.511(Topo = 2)) * exp(0.037 (tem) * exp(−0.007(rain)) *exp(0.02(hum).

The coefficients have an additive effect on the log(y) scale, and the IRR/odd ratio has a multiplicative impact on the y scale.

*The fitted Poisson regression model is*

. Topo = scale of Topography of the region

. Temp = Temperature

. Rain = amount of rainfall

. Hum = humidity.

Table 2 shows that the odds ratio for Top = 2 is 1.667 times the odds ratio for the reference group, Top =3. Similarly, the odds ratio for Top =1 is 0.665 times the odds ratio for the reference group, holding the other variables fixed. The percent variation in the odds ratio of incidence is 4% for every unit increase in temperature and 2% in humidity. The results in Table 3 show that the increase in rainfall, temperature, and humidity was significant to the malaria incidence (value of *p* <0.05). This confirms that rainfall raises parasitic thickness shortly after the start of the period. Malaria incidence is closely linked with temperature and humidity.

**Table 2:** Poisson regression results of covariates.

Parameter	Β	Std. Error	95% Wald CI		Testing of Hypothesis			Exp (β)	95% Wald C.I for Exp (B)	
			lower	Upper	Wald chi-square	d.f	Sig		Lower	Upper
Intercept Topo-1 Topo-2 Topo-3 Temp Rain Hum	4.001 −0.408 0.511 0^a^ 0.037 −0.007 0.021	0.0519 0.0115 0.0092 0.0007 0.0002 0.0006	3.899 −0.431 0.493 0.036 −0.007 0.020	4.102 −0.386 0.529 0.039 −0.007 0.022	5948.733 1269.080 3107.226 2710.042 1968.333 1335.613	1 1 1 1 1 1	<0.001 <0.001 <0.001 <0.001 <0.001 <0.001	54.639 0.665 1.667 1 1.038 0.993 1.021	49.357 0.650 1.637 1.036 0.993 1.020	60.486 0.680 1.697 1.039 0.993 1.022

*Topo = Scale of Topography of the region; Temp = Temperature; Rain = amount of rainfall; Hum = Humidity*.

**Table 3:** Autoregressive distributed lag (ARDL) model Results.

Variable	Coefficient	Std. Error	t-Statistic	Prob.
CASES(−1)	0.609762	0.159024	3.834405	0.0007
CASES(−2)	0.296420	0.180387	1.643246	0.1115
HUMIDITY____	−0.313694	11.41132	−0.027490	0.0320
RAINFALL__MM_(−1)	6.300892	2.973406	2.119082	0.0431
TEMPERATURE	56.17454	19.48938	2.882316	0.0075
R-squared	0.695569	S.D. dependent var		935.2815
Adjusted R-squared	0.641206	Akaike info criterion		15.65335
S.E. of regression	560.2282	Schwarz criterion		15.92271
Sum squared residue	8,787,957.	Hannan–Quinn criter.		15.74521
Log-likelihood	−260.1070	Durbin–Watson stat		2.174849

The statistical results of autoregressive distributed lag (ARDL) models is shown in Table 3, which shows the significant results of different variables. The results include an estimate of the coefficients of the model parameters, their standard error, t-test, and, finally, its significance values. The significance values for humidity, temperature, and rainfall are 0.0320, 0.431 and 0.0075, respectively. Hence, for all the three factors, the value of p or the significance value is less than 0.05; therefore, the results of the model are significant.

The results of the model also contained various measures, i.e., adjusted R-square, standard error of regression, residual sum of square, log likelihood, and Durban–Watson test, and the significance value for the factors. The value of R-square and adjusted R-square is quite high, which shows that the model is a good fit. Similarly, the Durban–Watson test proves that there is autocorrelation among the residuals.

## Discussion

In the current study, we analyzed the secondary data from the different hospitals of the Bannu district, Khyber Pakhtunkhwa, Pakistan. We observed 175,198 malaria cases in the study location. We conducted a time series analysis on 5 years of secondary data (2013 to 2017) and found that the relationship between malaria trends changes with respect to various climatic factors. To our knowledge, this is the first study in the region that used the fitted Poisson regression model to investigate the impact of temperature, humidity, and rainfall on the progression of malaria. The prevalence of vector-borne diseases can be effectively reduced through active and passive diagnosis (Zoghi et al., 2012). Accurate evaluation of malaria incidence can also help scale up control interventions and malaria surveillance in Pakistan (Yasinzai and Kakarsulemankhel, 2008). Previous studies have examined the epidemiological trends of malaria over several years. For example, a study conducted in Nsanje District, Malawi utilized trend analysis and time series analysis to predict the incidence of malaria in the district (Gondwe et al., 2021). Another study from Kumasi also used the time series model to forecast the future incidence of malaria (Anokye et al., 2018).

Malaria is one of the leading causes of morbidity and mortality in Pakistan (WHO, 2022). The prevalence of malaria in Khyber Pakhtunkhwa has been reported since the late 1970s. In the 1980s, the prevalence was high, while low prevalence was observed in the early 1990s (Rowland et al., 2002). This relatively high malaria prevalence that extended from Swat and Chitral in the north to Mardan, Malakand, Swabi, Khyber, and Mohmand along the western border with Afghanistan was seen in the late 1990s (Kazmi and Pandit, 2001). A primary study showed that the prevalence of malaria was high in Bannu district (Qureshi et al., 2020).

Climatic conditions have a great link with vector-borne diseases because these set the limits on the geography and seasonality of transmission, which is reflected in the distinct and often predictable seasonal distribution of vector-borne diseases (Ogden, 2017). Due to global warming of approximately 2°C, climatic changes involve increasing temperatures, changes to geographic patterns of rainfall, increasing climate variability, increasing frequency and severity of extreme weather events, and increasing humidity and precipitation, leading to more frequent extreme heat and precipitation events resulting in milder and shorter winters and summers to be warmer and longer. Elevation to higher degrees due to changes in climate and global warming causes greater changes, with extreme heat events, daily-scale precipitation extremes, and a further increase in annual precipitation (Romero-Lankao et al., 2014). These climate changes facilitate the emergence and transmission of exotic vector-borne diseases in numerous ways. Warmer temperatures, higher humidity, and increased precipitation facilitate the lifecycle of exotic mosquitoes by supporting larval development and survival and extending adult lifespan, thus increasing overall population size (Romero-Lankao et al., 2014).

There are widely three anticipated threats (Ogden et al., 2013). First, the danger from endemic vector-borne sicknesses may also increase due to long-term temperature changes and rainfall patterns. Mosquito-borne illnesses may also result in greater epidemics, as the vectors and pathogen transmission cycles increase with growing climate variability and intense weather activities. Second, vector-borne illnesses may shift their geographic degrees poleward (or to higher altitudes in the mountains) into areas with low or zero incidence. Third, climate exchange may also increase the threat of the established order of ‘uncommon’ tropical/subtropical vector-borne diseases through abrupt changes in climatic suitability, transmission from endemic areas, and international migration (Ogden, 2017).

Malaria occurrence is narrowly related to temperature. It affects malaria transmission, maybe because of two causes: either the temperature is too low to stop the virus and vector growth or too high that it causes an enlarged expiry of the parasite. A low temperature of 16^°^C controls bloodsucker (parasite) growth and stops the growth of the vector in its liquid levels. At 17^°^C, bloodsuckers mature but are not speedily sufficient to source an endemic. The result of the present study shows that, with changes in degrees of temperature, malarial prevalence has been affected. A temperature increase has been considered to one of the reasons for malaria transmission in Europe (Fischer et al., 2020). Global warming due to human activities also increases the incidence of malaria (Caputo and Med, 2016). A Chinese study found that each 5^°^C temperature compared to 10^°^C increase increases 175% of malaria cases in China and assumed high temperatures with humidity and rainfall as an influencing factor for malaria transmission (Liu et al., 2021).

The result of the present study shows that rise and fall in humidity affect the incidence of malaria. **Humidity** is linked to rainfall, and it raises the lifecycle of parasites, giving them extra chances to transfer malaria poisons from one person to another person. It has been observed that the summer rainy season increases malaria incidence. Malaria significantly increases due to temperature, moisture, and humidity (Herekar et al., 2020). Relative humidity measures the amount of water vapor in the air, with 0% indicating dry air and 100% indicating fully saturated, humid air. Relative humidity affects the malaria transmission cycle by influencing mosquito behavior and survival.

Rains delivered good propagation positions for the parasite vectors (Patz and Reisen, 2001). Malaria incidence increases with an increase in rainfall. According to the weather forecasting regional department, it commonly records one of the maximum rainfall quantities in the area. As the parasite’s numbers increased, the spread of infection was boosted, and thus, the increase in the number of parasites. Additionally, heavy rainfall may have increased humidity, fostering the existence of parasites. Earlier, in an African study, the seasonal dynamic role of rainfall was identified. The winter rainy season (December to February) increased the malarial cases in the study area (Adeola et al., 2019). There is also an impact of topography on malaria incidence. In our study, through Poisson regression models, we found that there is a significant relation with topography. Earlier, a study reported that malaria incidence was high in lowland areas as compared to highland areas (Atieli et al., 2011). Geographical highland factors influence vector survival at different elevations, which, in turn, influences vector abundance and the possibility of malaria transmission (Minakawa et al., 2002). These precise models for forecasting malaria and considering the effects of terrain and climate will be utilized to identify high-risk locations for prompt intervention in future by concerned authorities and researchers. Climatic factors and topography significantly affect the malaria incidence, highlighting the importance of consideration of climatic factors in malaria control strategies.

There were certain limitations in the study. This study did not find a link between socioeconomic determinants with malaria. However, the researchers are planning to conduct a study focusing on the socioeconomic determinants of malaria in the region. Secondary data are limited to certain information. Another limitation of the study was the absence of information on imported cases and non-reported cases of malaria to the health facilities, which may affect the overall incidence of malaria in the region. This study included a short time series, i.e., 2013–2017; therefore, there is a need for further research to validate our findings. There is also a need of potential for future research to explore lagged effects and their impact on malaria incidence. Finally, the Poisson regression model is only limited to a discreet dependent variable. Other robust regression models can be applied if the basic assumptions are violated.

## Conclusion

The results of the Poisson regression model to the vector-based disease, i.e., malaria incidence, were due to different factors, including rainfall, topography, humidity, and temperature, in the study area. The finding of this study explored the fact that the temperature, humanity, rainfall, and topographic variables have a significant association with the incidence of malaria. There is a need for future studies with longer time series data to enhance the robustness of the findings. Our findings could help in designing malaria control strategies in the region. Effective surveillance and timely responses can reduce malaria incidence. Local health administrations need to ensure clean and well-maintained drainage systems to prevent mosquito breeding grounds. Proper modeling for predicting malaria is essential. It can help identify high-risk areas where authorities can intervene.

## Data availability statement

The original contributions presented in the study are included in the article/Supplementary material, further inquiries can be directed to the corresponding authors.

## Author contributions

IH: Conceptualization, Methodology, Writing – original draft. ZM: Conceptualization, Formal analysis, Supervision, Writing – review & editing. GK: Writing – review & editing. BK: Data curation, Investigation, Conceptualization, Formal Analysis, Methodology, Supervision, Writing – original draft. BA: Data curation, Methodology, Conceptualization, Investigation, Supervision, Writing – review & editing. JS: Data curation, Investigation, Formal analysis, Writing – original draft. AS: Supervision, Validation, Formal analysis, Methodology, Project administration, Conceptualization, Funding acquisition, Writing – original draft. MN: Data curation, Formal analysis, Software, Conceptualization, Validation, Visualization, Writing – review & editing. JX: Validation, Writing – review & editing. HX: Supervision, Visualization, Writing – review & editing.
