# Supplementary material for: Modeling the effect of climatic conditions and topography on malaria incidence using Poisson regression: a Retrospective study in Bannu, Khyber Pakhtunkhwa, Pakistan
Source: Front Microbiol. 2024 Jan 15;14:1303087. doi: 10.3389/fmicb.2023.1303087 (PMC10822983; doi:10.3389/fmicb.2023.1303087)
Supplement: Supplementary file 1 [file Data_Sheet_1.docx]

**Responses to the Editors and Reviewers**

**Dear Editor-in-Chief,**

**Frontiers in Microbiology**

Thank you and the reviewers for the favourable recommendations to our manuscript, submitted to the **Frontiers in Microbiology**, entitled " **Modeling the effect of climatic conditions and topography on malaria incidence using Poisson regression: A retrospective study in Khyber Pakhtunkhwa Pakistan**". We are pleased to have the opportunity to submit our 3^rd^ time revision. Point by point, response to the editors and reviewers' comments are below. Changes are in blue in the revised manuscript.

Point to point responses of the reviewers has been added. Changes has been shown in blue.

**Guest Editor Comments and responses to the guest editor**

The manuscript lacks clarity and a coherent structure. The introduction should clearly establish the public health problem and research objectives, and the content should be organized to align with these objectives. (Please refer to Lines 46-115)

**Response:** Thank you for the detail comment. According to your kind suggestion we have revised the introduction and make it clear. Also, we have linked the introduction with the objectives.

Several statements in the manuscript lack proper references.

**Response:** We have added the missing references in our manuscript.

The authors should ensure that all factual claims are supported by relevant and up-to-date literature.

**Response:** Thank you for the comment. We have added the updated literature. (Lines 46-115)

The methods section requires significant revision for clarity. The data collection process, including participants and sources of data, should be described in detail.

**Response:** Thank you. We have clarified our methods and described the collection processes. (Lines 127-147)

The statistical analysis needs to be adequately explained. It's unclear why logistic and Poisson regression were used, and the choice of these methods should be justified. Additionally, details about model fitting and selection are essential.

**Response:** About the statistical models, the usual simple correlation coefficient is not used here because the data as asymmetric in case of rainfall. We have applied Poisson regression model as the dependent variable is a discreet that is malaria positive cases in each month is just a count variable. We are using this with the understanding that regression models are better to establish the relationship between response variable with related explanatory variables as compared to calculate simple correlation.

The manuscript mentions data quality control and validation processes, but these need to be explained more thoroughly. How data quality was ensured should be clearly documented.

Response: Data was checked by the Dr Zafar Mehmood who is an experienced biostatistician and Dr Ijaz ul Haq. They checked data for issues such as duplication, data entry errors, and other data quality issues. (Line 142)

The results and discussion sections should be improved to better summarize the findings and align with the methods and objectives of the study. There is a need for a more detailed presentation of the results and their implications.

**Response:** Thank you for the comment. We have revised our results section and implications has been added Line#305-309.

The manuscript mentions ethics considerations but lacks an approval number. This approval should be obtained and included in the paper, and the ethical aspects of data collection should be described.

**Response:** Institutional review board of The University of Agriculture, Peshawar approved this study. The ethical approval number in the manuscript has been added (Line 130).

**Responses to Reviewers:**

***Reviewer 4***

Modeling the effect of climatic conditions and topography on malaria incidence using Poisson regression: A retrospective study in Khyber Pakhtunkhwa Pakistan

Introduction

Paragraph 1 (lines 46-64): writing is disjointed and would benefit from editing for clarity. The focus should be on malaria as a global burden of disease.

**Response**: Malaria as a global burden of disease has been given in the first and 2^nd^ paragraph now.

**Response:** Thank you for the comment. Literature regarding “malaria as a global burden of disease” has been given line (Line 47-64). The mentioned paragraphs has been revised.

Paragraph 2 (line 65-69): references are needed to support factual statements.

**Response:** Thank you we have included the reference. (Line 76-81)

Paragraph 3

Lines 71-73: edit for clarity. The phrase “...calamitous complications and can thrust drastic and far73 reaching consequences within a community…” is vague and subjective.

**Response**: We have revised it to “In severe cases, malaria leads to calamitous complications, which have significant and widespread consequences in a community.” (Line 83-85)

Lines 80-82: Environmental factors should be discussed together with previous paragraphs.

**Response:** Thank you. We have changed it accordingly. (Line 72-75)

Paragraph needs to narrow it’s focus - rather than providing a review of malaria, the discussion should be more closely centered on the goals/objectives of this study.

**Response:** Thank you for the comment. This paragraph provides a review of the problem, and the discussion about the objectives are in the last paragraph.

Paragraph 4

I suggest starting the introduction section with this paragraph. Establish the public health problem first.

**Response:** Thank you we moved this paragraph to the start of introduction. (Line 45-52)

Paragraph 5

Lines 94-95: “a six-fold increase in P. falciparum malaria has been observed…”. Observed relative to what? Please clarify.

**Response:** P. falciparum malaria has been observed in the last decade. We have revised it. (Line 47)

Lines 96-97: factual statement needs a reference.

**Response:** Thank you. We have added the reference according to the suggestion.

Paragraph 6:

References used in this paragraph are decades old and should be updated to reflect a more current understanding of malaria in Pakistan.

**Response:** Thank you, we updated the reference. ( Line 48-52)

Paragraph 7:

Lines 108-112: needs editing for clarity. I recomend merging with the goals/objectives paragraph at the end.

Response: We have merged it to the final paragraph. (Line 102-106)

Paragraph 8:

Line 113: What does “malaria is naturally unbalanced” mean?

**Response:** Thank you. It means unusual distribution. We revised our statement as follows. (Line 106-107)

Line 114-115: Why is this phrase included here? “But pregnant ladies and kids under five years are a high threat.” Are these variables included in the analysis? References need to be provided for factual statements.

**Response:** Thank you. It means the high vulnerable groups to malaria. As we haven’t included it in the analysis, we omit this.

This paragraph needs to be re-written to clearly describe the knowledge gaps and the goals/objectives addressed in this study.
**Response:** Thank you we revised it accordingly. (Line 107-114)

METHODS

Study Area and Participants: This section needs to be rewritten for clarity and to better fit with the objectives of this study. For example, participants are not described. In general, the information presented is disjointed and unclear.

**Response:** As for the participants, we collected secondary data from health facilities and other offices. Our data included the incidence of malaria cases, topography, and climatic factors. In the secondary data, we did not obtain the sample characteristics. These decisions were made in line with our study objectives, as we aimed to assess the impact modelling of malaria incidence on climatic factors and topography.

Lines 136-138: Include the reference numbers for the IRB approval.

**Response:** Thank you. We include the IRB number. (Line #130)

Data Collection:

Line 140: What is area-wise data?

**Response:** Thank you for the comment. It means data was obtained from different neighbourhood councils, union councils and tehsil wise data.

Line 141: reference for census data is needed.

**Response:** Thank you we have provided it.

Lines 141-143: include reference for the environmental data.

**Response:** Environmental data was obtained from the concern office on the request. (Line 133)

Line 143: “local metrological office Bannu District, Khyber Pakhtunkhwa, Pakistan”. This sentence is confusing - what is it refering to?

**Response:** we replaced it by “Regional Meteorological Office”. We obtained data from it. (Line 135)

Lines 143-145: “Rain and humidity data throughout the months were documented, while the temperature measurements in degrees celsius were recorded” is vague and needs to include specific details (location, time frame, etc.). It is not reproducible as written. How were they documented? Where did this information come from (reference needed)?

**Response:** We have revised the whole paragraph. We obtained secondary data from the concern department. Letters were sent to them for the data. (Line 134-137)

Lines 145: “The figures of malarial fever persons were gathered…”. This is vague and needs to be substantially expanded upon.

**Response:** We revised it. Thank you. (Line 137-139)

Lines 150-151: “Experienced statisticians and researchers checked the quality of data”. How was it clarified? This sentence is subjective as written.

**Response:** Data was checked by the Dr Zafar Mehmood who is an experienced biostatistician and Dr Ijaz ul Haq. They checked data for issues such as duplication, data entry errors, and other data quality issues. (Line 142)

Lines 151-153: Case definition needs to be more detailed. What is the source of this definition? Were there additional exclusion/inclusion criteria?

**Response:** For this study, the case definition of malaria included individuals with a confirmed record of malaria who were not hospitalized, as well as individuals with hospitalization records indicating a diagnosis of malaria. Malarial parasite blood smear test was conducted to diagnose malaria in patients.

All the incidence data for the respective years was included in the study. (Line 142-145)

Statistical Analysis: This section does not adequately describe the analysis. Why were logistic and poisson regression used? In the previous section, incidence rates are described, which means neither of these methods are appropriate here. How were models fit?

**Response**: Thank you for the comment. We made mistake by mentioning logistic regression. We use Poisson regression for the modelling. We have also provide the details in the supplementary file 1. (Line 152)

We have applied poisson regression model as the dependent variable is a discreet that is malaria positive cases in each month is just a count variable. We are using this with the understanding that regression models are better to establish the relationship between response variable with related explanatory variables as compared to calculate simple correlation.

RESULTS/DISCUSSION:

These sections do not adequately summarize the results and, as written, does not reflect the information provided in the methods section. The entire sections need to be edited for clarity and to more accurately reflect the methods and goals/objectives.

**Response:** We have revised our discussion and necessary text has been added.

**Reviewer 5**

The language of the manuscript is not understandable at several places. Many statements are erroneous. There is a need for such studies from Pakistan. Unfortunately, the authors could not analyze the relationship between climatic factors and malaria cases in a simple and effective way. The correlation coefficient would have given valuable output. What is the lag period between rainfall and the occurrence of cases? Further, the time series of only five years is inadequate for determining such a relationship.

**Response:** Thank you to the respected reviewers for the details comments. Time series model was used for monthly data as we know that time series model is usually giving valid results if used for larger period of time. We have thus used the time series model for 48 months with a lag of month.

The usual simple correlation coefficient is not used here because the data as asymmetric in case of rainfall. We have applied poisson regression model as the dependent variable is a discreet that is malaria positive cases in each month is just a count variable. We are using this with the understanding that regression models are better to establish the relationship between response variable with related explanatory variables as compared to calculate simple correlation.

Earlier studies also conducted short time series models for 5 years such as “Epidemiological Trends of Malaria in Five Years and under Children of Nsanje District in Malawi, 2015–2019”, “Time series analysis of malaria in Kumasi: Using ARIMA models to forecast future incidence”.

Thankful to the reviewer for all valuable suggestions and most of the suggestions are incorporated in the revised manuscript.
Introduction part is too lengthy.

**Response:** Some text has been removed. Thank you for the comment.

Detailed comments are given below:
Line 57-59: The statements are erroneous e.g. The cause of all VBDs are arthropods that transmit viruses---. Dengue and malaria are not fomites

**Response:** Thank you for the comment. We have revise it for the clarity. (Line 67-70)

The cause of vector-borne diseases (V.B.D.s) are arthropods that transmit viruses among humans. Example of V.B.D.s are dengue and malaria (mosquito-borne), lyme disease (tick-borne), and leishmaniosis (transmitted by sandflies).
Line 60-61: It is not known that arthropods change their body temperature depending upon climate.

**Response:** Thank you for the comment. We have remove this statement.
Line 66-67: It is not correct that with increase in elevation malaria also increases.

**Response:** Thank you for the comment. We revised our statement according to the previous literature. “Topography also has a high role in spreading vector-borne diseases. Incidence of Malaria and topography have a positive relationship, i.e., the risk of malaria increases in highlands compared to lowlands.” (Line 76-81)
Line 82 ‘growing the probabilities of malaria diffusion’ is not clear.

**Response:** Thank you for the comment. It should be malaria transmission. ( Line 107)

“Higher temperatures can reduce the extrinsic incubation period, potentially increasing the likelihood of malaria transmission.”

**Response:** It has been taken from the literature.
Line 94: ‘Destruction of malaria is widspread’ meaning not clear.

**Response:** According to the other reviewer we have to put this paragraph in the start. We revised this sentence as “In Pakistan, malaria is endemic, affecting millions of lives per year.”
Line 105: ‘malaria is stable and variable with climate change’. If it is stable malaria, it should change much with climate change.

**Response:** We changed and revised the statement as “In K.P., malaria incidence is in alarming situation [Incidence of Malaria in Khyber Pakhtunkhwa Pakistan- A Meta-Analysis]. There is a connection between malaria and climate change, as the outbreak of malaria has been observed in the aftermath of heatwave in the past (Pakistan’s lessons learned on climate change, malaria, and vector-borne disease transmission) URL: . chrome-extension://efaidnbmnnnibpcajpcglclefindmkaj/https://www.dmc.gov.pk/wp-content/uploads/2023/08/Pakistan-Case-Study.pdf).”
Line 115: ‘Pakistan is the lone country where malaria is naturally unbalanced’? sentence not clear.

**Response:** We have revised it as “ In Pakistan, malaria exhibits an unusual distribution.”
Line 135-136: ‘Total precipitation calculated in 2013 is 15.8 mm as compared to 0.3 mm in 2012’ does not appear correct. Whether the precipitation is only for winter?

Response: This old data which has been omitted from the methodology. We have shown rainfall, humidity and temperature for the whole year in results ( Table 1).

Line 149-151: ‘These data were evaluated per thousand individuals of the populace and were more evaluated through the formula Incidence for the clusters of individuals under training’ the meaning is not clear.

**Response:** Thank you for the comment. This sentence was regarding the incidence but due the comment from other reviewers, we removed this. Thank you for highlighting.
Line 181: ‘Rainfall consequently increases parasitic density in the area’ rainfall does not increase parasite density.

**Response:** Thank you for the comment. We revised the statements. We also found that with increase in the rainfall, malaria incidence increases.
Line 200-201: ‘The result indicates that rise and fall in humidity affect malaria incidence’. The sentence is vague.

**Response:** Thank you we omit that sentence.

Line 259: 61975 cases of malaria were observed in the study location while the methodology section mentions 175198 ( line 172).

**Response:** Thank you. The total cases are 175198. We corrected it in discussion. (Line 161 and 233)

Thank you
